# Pushing the hybrid approach to the edges, three stories in one: a case report

**DOI:** 10.1093/ehjcr/ytae333

**Published:** 2024-07-11

**Authors:** Sotirios Dardas, Petros Dardas, Nikolaos Mezilis, Dimitrios Tsikaderis, Theodoros Kofidis

**Affiliations:** Department of Interventional Cardiology, St Luke’s Hospital, Panorama, Thessaloniki, 55236, Greece; Department of Cardiology, University Hospitals of Derby and Burton NHS Foundation Trust, Royal Derby Hospital, Uttoxeter Rd, Derby, DE22 3NE, UK; Department of Interventional Cardiology, St Luke’s Hospital, Panorama, Thessaloniki, 55236, Greece; Department of Interventional Cardiology, St Luke’s Hospital, Panorama, Thessaloniki, 55236, Greece; Department of Interventional Cardiology, St Luke’s Hospital, Panorama, Thessaloniki, 55236, Greece; Department of Cardiac, Thoracic and Vascular Surgery, National University Heart Center, Singapore City, Singapore

**Keywords:** Mitral annular calcification, Severe mitral regurgitation, Minimally invasive endoscopic procedure, Bioprosthetic valve degeneration, Prosthetic valve migration, Valve in valve procedure, Case report

## Abstract

**Background:**

Mitral annular calcification (MAC) is common in the elderly. Extensive calcification has been historically challenging for the cardiac surgeons, with traditional surgical approaches carrying significant risks. Less invasive approaches have recently been explored in an attempt to reduce this risk.

**Case summary:**

We report the case of a 75-year-old woman who presented with recurrent pulmonary oedema, due to severe MAC and mitral regurgitation. Her past medical history included bioprosthetic aortic valve replacement 5 years ago. Given the extensive MAC and the patient’s frailty, a minimally invasive hybrid approach with direct implantation of a transcatheter balloon expandable Sapien 3 valve was selected to manage her. Although the post-surgical result was initially excellent with elimination of the mitral regurgitation, the patient’s post-operative course was marked by two serious complications, namely, acute severe aortic regurgitation, due to rupture of the bioprosthetic valve’s right cusp, and severe paravalvular leak of the Sapien valve, due to posterior migration towards the left atrium. These were managed successfully with emergency valve-in-valve implantation using the ‘double chimney’ technique for the bioprosthetic aortic valve, as well as transeptal valve-in-valve implantation of a 2nd Sapien valve in the mitral valve, which sealed the gap between the 1st Sapien and the calcified mitral annulus.

**Discussion:**

This case illustrates a less invasive approach for the management of severe MAC. Complications can still occur in this high-risk group of patients, and therefore, such cases should be managed with close collaboration between cardiac surgeons and cardiologists, in centres with high expertise.

**Learning points**

- Mitral annular calcification is frequent in the elderly. Traditional surgical approaches are associated with significant life-threatening risks.
- A minimally invasive hybrid approach with direct implantation of a balloon expandable valve is feasible and overcomes several of the surgical risks.
- These procedures should be performed in centres with high expertise, where all potential complications can be managed timely and efficiently.

## Introduction

Mitral annular calcification (MAC) is common in the elderly (prevalence 8–42%),^1^ characterized by progressive mitral annular calcific deposition. Although the exact aetiology is not well understood, contributing factors include aging and altered calcium metabolism, such as in renal disease.^1,2^ It is linked with increased cardiovascular morbidity/mortality and frequently associated with significant mitral stenosis or regurgitation.^2^

## Summary figure

**Table:** 

Timeline of critical events	
Day 0	Patient presented to our Heart Centre for evaluation of her mitral valve disease. Investigations revealed severe MAC with severe regurgitation.
Day 4	Heart Team decision for minimally invasive approach, due to the high risk and patient’s frailty.
Day 10	Minimally invasive hybrid procedure with direct implantation of a transcatheter balloon expandable Sapien 3 valve with good outcome.
Day 17	Acute deterioration with severe pulmonary oedema requiring intubation and haemodynamic support. Transoesophageal echocardiogram (TOE) demonstrated acute severe aortic regurgitation (AR) due to rupture of the bioprosthetic valve’s right cusp. Emergency transcatheter aortic valve implantation computed tomography (CT) performed, followed by emergency aortic valve-in-valve procedure with ‘double chimney’ technique, resulting in immediate haemodynamic improvement.
Day 27	Further deterioration with pulmonary oedema and haemolysis. TOE demonstrated severe paravalvular leak (PVL) of the Sapien valve, due to posterior migration towards the left atrium.
Day 30	Percutaneous implantation of 2nd Sapien 3 valve on a lower position, which sealed the gap between the 1st Sapien and the calcified mitral annulus, resulting in significant reduction of the PVL.
Day 35	Implantation of dual chamber pacemaker for episodes of intermittent complete heart block.
Day 55	Patient discharged in good state following a period of rehabilitation.
12 months post-discharge	Patient remains well and asymptomatic.

Extensive calcification historically posed a challenge for the cardiac surgeons, with traditional surgical approaches, such as annular decalcification and reconstruction carrying significant risks, including rupture at the atrioventricular junction, PVL, left circumflex artery injury, and embolism. Hence, these patients have frequently been deemed inoperable. Nevertheless, left untreated prognosis is poor.^2^

This article describes a case of severe MAC and regurgitation which was managed with a minimally invasive hybrid approach, aiming to overcome those risks. The post-operative course was marked by serious complications, the management of which is described here.

## Case presentation

A 75-year-old woman presented to our Heart Centre for evaluation of mitral valve disease. Past medical history included a surgical aortic valve replacement with a 21 mm bioprosthetic Mitroflow valve (Sorin, Milan, Italy) for severe aortic stenosis 5 years prior. She had recently been treated elsewhere for recurrent pulmonary oedema, during which severe MAC with severe regurgitation was diagnosed but was deemed inoperable. Examination on the current admission revealed a loud pansystolic murmur at the apex extending to the axilla, and the patient was euvolaemic.

**Figure 1:**
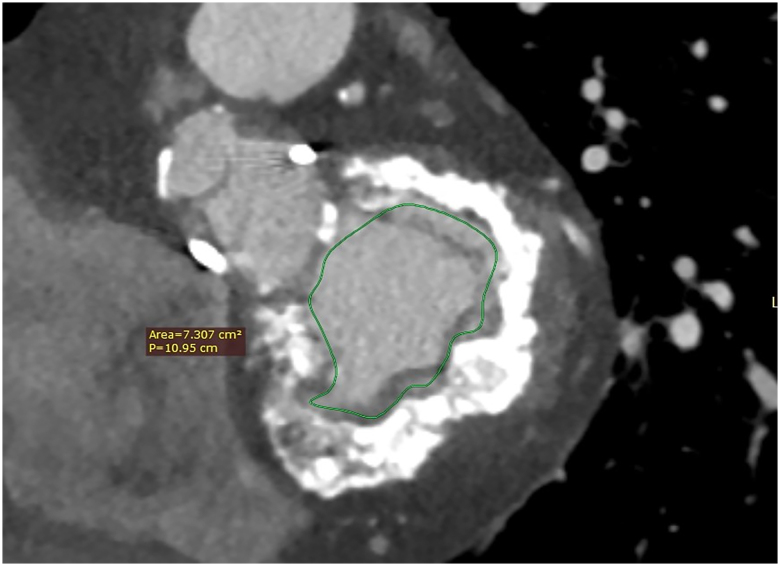
Initial cardiac computed tomography—mitral annular calcification. Cardiac computed tomography demonstrating the almost circumferential mitral annular calcification, as well as the mitral valve annular area. 100 × 138 mm.

TOE demonstrated severe MAC with severe regurgitation (*Video 1*). The previous bioprosthetic Mitroflow aortic valve appeared moderately stenotic and mildly regurgitant. Cardiac CT confirmed extensive and almost circumferential MAC (*Figure 1*). Invasive coronary angiography showed unobstructed coronaries.

**Figure 2:**
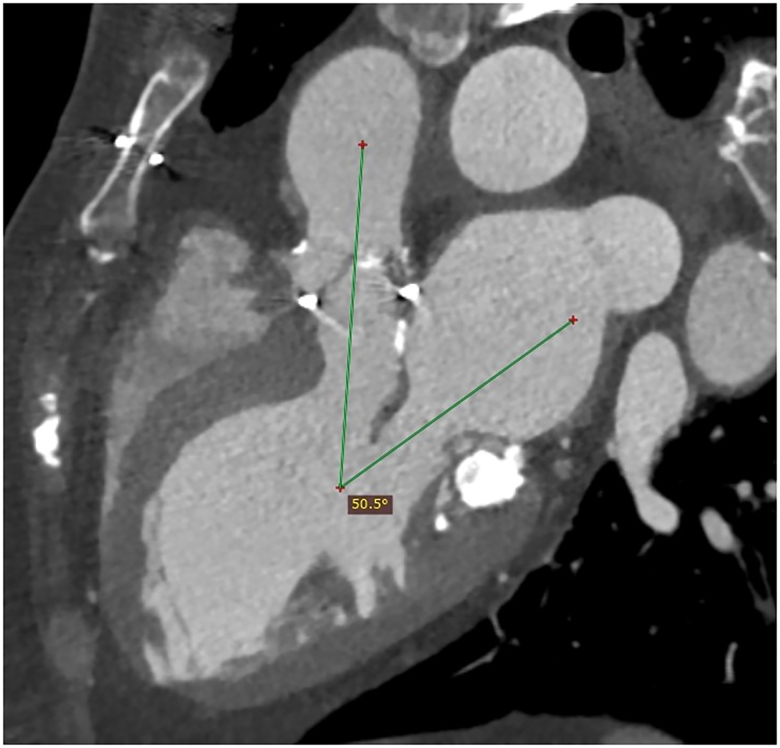
Initial cardiac computed tomography—aorto-mitral angle. Cardiac computed tomography demonstrating acute aorto-mitral angle, posing increased risk for left ventricular outflow tract obstruction. 115 × 120 mm.

Following Heart Team discussion, the risk of conventional surgery was considered prohibitive, given the extent of MAC and the patient’s frailty. Percutaneous transeptal delivery of balloon expandable valve was not chosen due to inhomogeneous calcium distribution and risk of left ventricular outflow tract obstruction (LVOTO), related to acute aorto-mitral angle (*Figure 2*). To reduce that risk whilst achieving optimum outcome, a minimally invasive hybrid approach with direct implantation of a transcatheter balloon expandable valve was favoured. This was performed endoscopically, via a 4 cm right mini-thoracotomy, on femoro-femoral cardiopulmonary bypass (CPB) and beating heart, guided by 3D camera. The anterior leaflet was resected to avoid LVOTO, prior to implantation of a 29 mm Sapien 3 valve (Edwards Lifesciences, Irvine, CA, USA). Due to enormous amount of calcium, only two sutures could be placed anteriorly (see Supplementary material online, *Video S1*). The post-surgical result was initially very good with no LVOTO and mild PVL, which was deemed acceptable given the circumstances (*Video 2*).

**Figure 3:**
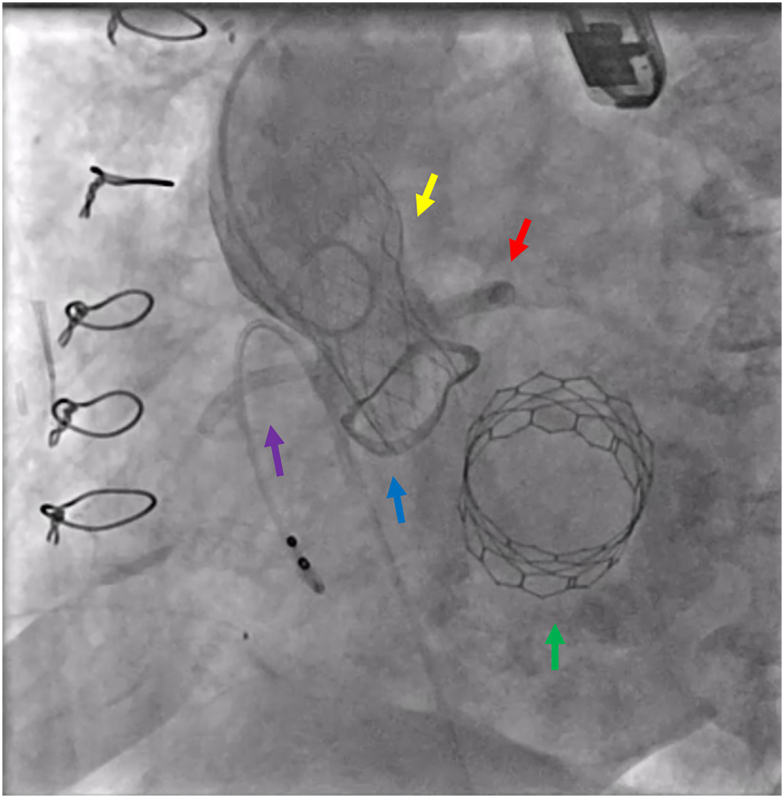
Transcatheter aortic valve implantation. Fluoroscopy showing deployed 23 mm Evolut Pro Plus (yellow arrow), within the Mitroflow valve (blue arrow), left main stem stent (red arrow), right coronary artery stent (purple arrow) and Sapien 3 valve sitting on the calcified mitral annulus (green arrow). 120 × 118 mm.

The patient remained stable for 7 days, after which she deteriorated with severe pulmonary oedema requiring intubation and inotropic/vasopressor support. TOE demonstrated severe AR due to rupture of the bioprosthetic valve’s right cusp (see Supplementary material online, *Video S2*). Urgent CT scan was performed to determine the technical feasibility of a transcatheter aortic valve-in-valve implantation, and she was moved immediately to the catheterization laboratory, where a 23 mm Evolut Pro Plus valve (Medtronic, MN, USA) was implanted within the Mitroflow aortic valve, resulting in immediate haemodynamic improvement. Of note, both coronary ostia originated very low to the Mitroflow valve and the sinuses of Valsalva were narrow. This anatomy creates the risk of the bioprosthetic valve leaflets potentially occluding the coronary ostia, and therefore, as a precaution, we positioned undeployed stents within both the coronary ostia prior to the Evolut valve deployment. After deploying half of the valve, the patient developed global ischaemia on electrocardiography, and hence, we deployed both the stents simultaneously with the valve, using the so called ‘double chimney’ technique. Both stents were subsequently post-dilated to achieve optimal result (*Figure 3*). It is important to note here that the choice of an Evolut rather than Sapien valve was made for several reasons. Given the small size of the Mitroflow aortic valve (21 mm), the applicable Sapien would have been a 20 mm intra-annular valve, which could result in high residual trans-aortic gradient. Besides, both the coronary ostia laid very low, which necessitated their protection irrespective of the chosen valve. Finally, it was felt that the use of balloon expandable valve could potentially deform the surgically implanted Sapien valve in the mitral position.

The patient remained stable during the following days, however, progressively deteriorated again with pulmonary oedema and haemolysis. Another TOE revealed severe PVL of the surgically implanted Sapien valve in the mitral position, due to late posterior migration towards the left atrium, at the points where sutures could not be placed (*Video 3*).

**Figure 4:**
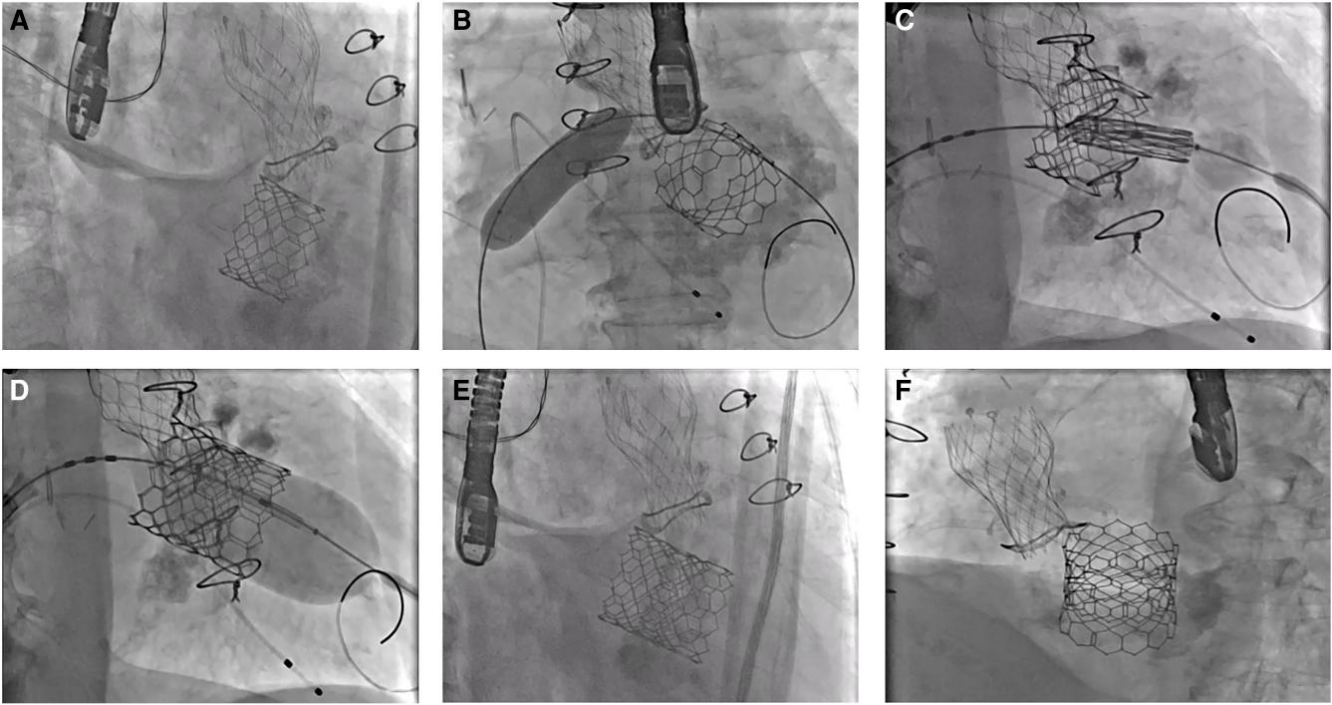
Sapien in Sapien implantation. Fluoroscopic appearances demonstrating step-by-step implantation of the 2nd Sapien valve. (*A*) Pre-implantation, (*B*) septostomy with 14 mm balloon, (*C*) valve positioning, (*D*) post-dilatation and (*E* and *F*) final appearances. 87 × 164 mm.

Following Heart Team discussion, a 2nd 29 mm Sapien Ultra valve was implanted transeptally on a lower position towards the left ventricle. This sealed the gap between the 1st Sapien and the calcified mitral annulus (*Figure 4*). The decision for this approach was driven by the prohibitive risk of a redo surgical procedure. In addition, the previous excision of the anterior mitral valve leaflet would prevent the initially considered risk of significant LVOTO.

Final TOE demonstrated significant reduction of the PVL from grade IV to grade I (see Supplementary material online, *Video S3*). The peak and mean LVOTO gradients increased to 34 and 18.5 mmHg, respectively, without any clinical consequence.

A pacemaker was implanted subsequently for intermittent complete heart block. This was felt to be an almost expected outcome, given the multiple interventions in close proximity to the atrioventricular node.

Psychological support was provided to the patient and her family throughout this very challenging time for them.

Following a period of rehabilitation, she was discharged in a good state after an eventful and prolonged admission totalling 55 days.

She remains well and asymptomatic 12 months following hospital discharge.

## Discussion

The management of patients with severe MAC remains extremely challenging, due to the risks highlighted previously. Over the last decade, less invasive techniques have been developed in an attempt to offer symptomatic improvement and minimize complications. In this direction, various techniques have been reported, which involve the implantation of transcatheter balloon expandable valves delivered either directly using a hybrid strategy involving CPB, cardioplegic arrest and left atriotomy or purely via the transcatheter route, facilitated by transfemoral/transapical access.^1,2,3^

Experience so far is limited, with evidence from randomized controlled trials lacking and most data being derived from small retrospective studies.^4^ The 30-day mortality of the percutaneous transcatheter valve in MAC in the Transcatheter Valve Therapy registry was 21.8%.^5^ In a recent multicentre prospective study, the MITRAL trial, the use of balloon expandable aortic transcatheter valves for selected patients with severe MAC was associated with sustained improvements in quality of life and heart failure symptoms, as well as stable valve function at 2 years of follow-up. Nevertheless, the mortality remained high (39.3%), also highlighting the comorbidity burden of these patients and need for better patient selection.^3^

Compared with pure transcatheter procedures, the hybrid approach seems to offer additional benefits. Apart from avoiding the risks of extensive annular decalcification, it facilitates resection of the anterior mitral leaflet, minimizing the risk of LVOTO.^2^ Moreover, various methods can be used to further stabilize the balloon expandable valves, most commonly sewing a felt strip around the valve and through pledgeted sutures directly onto the leaflet remnants, further reducing the valve migration and PVL risk.^1,2^ On the other hand, this approach is more invasive and requires CPB. In addition, other strategies in managing patients with severe MAC have been described in the literature. Although mitral transcatheter edge-to-edge repair has been reported to be safe and provide durable reduction of mitral regurgitation in carefully selected patients, these patients had less reduction in symptoms and higher 1-year mortality compared with patients with none/mild MAC, and so more research is required to identify patients who would benefit the most with this approach.^6^ Another approach that has been described is this of partial upper sternotomy, as a safe alternative in traditional mitral annular decalcification.^7^ This was deemed to be high risk in our patient, given the previous sternotomy and her frailty.

In our case, it is important to emphasize that although it is very difficult to postulate the exact causes of the complications encountered, these could at least in part have been related to the procedures themselves. Regarding the acute severe AR, we hypothesize that balloon overstretching, which was employed originally to adequately expand the surgically implanted Sapien valve in the heavily calcified mitral annulus, may have deformed the already moderately degenerated and in very close proximity Mitroflow aortic valve, leading to loosening and subsequently rupture of its right cusp. It is worth mentioning here that the Sorin Mitroflow valve has externally mounted bioprosthetic leaflets, which perhaps are more vulnerable to manipulations at nearby structures (such as the mitral valve), compared with a similar internally mounted valve that would have an annular ring and leaflet mounts protecting it. Another hypothesis is that elimination of mitral regurgitation with the subsequent increase in the left ventricular stroke volume could potentially have contributed to the accelerated bioprosthetic aortic valve degeneration. This in turn may have contributed to the subsequent Sapien mitral valve migration, via a mechanism of left ventricular and mitral annular stretching related to severe AR.

Of course, the inability to place enough sutures around the original Sapien valve played a role to its subsequent migration. Our original intention was to suture a felt strip around the valve; however, this was abandoned intra-operatively as the 29 mm Sapien appeared to fit well. Attempts to secure additional valve stabilization were unsuccessful, as it was impossible to place any further sutures to the extremely calcified annulus, especially posteriorly. This, together with the decision not to place a felt strip, were felt to be important contributing factors to the observed late valve migration. Apart from insecure device fixation to the mitral annulus, other proposed mechanisms of this complication in the literature are these of either device malposition or suboptimal device delivery.^8^

Our case is unique for many reasons. It presents one of the very few reported cases of hybrid implantation of a balloon expandable valve in the mitral position using a minimally invasive endoscopic approach on a beating heart as a redo operation. Despite the multiple serious complications experienced by the patient, the final outcome was favourable, highlighting the importance of being prepared to deal with all the complications that may be encountered when facing with similar situations.

To the best of our knowledge, this is the 1st case ever described where a 2nd Sapien valve was implanted to correct the complication of late valve migration and severe PVL in a patient with severe MAC.

This extreme case adds to the limited experience of using transcatheter balloon expandable valves in patients with severe MAC. Careful patient selection and planning is of utmost importance, as is close collaboration of the Heart Team. Flexibility in the decision-making process and innovative problem-solving are particularly important attributes of an effective multidisciplinary team, which can prove life-saving in challenging cases like this, when multiple complications occur. Such cases should be managed in centres with high expertise, where all the available techniques and equipment can be utilized, so that risk is minimized and all complications can be managed promptly to achieve favourable outcomes.

## Lead author biography


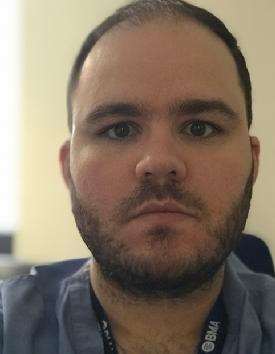


Dr Sotirios Dardas graduated from the Medical School of the Aristotle University of Thessaloniki, Greece, in 2014. He subsequently moved in the UK for his post-graduate training. He is currently a cardiology specialty trainee in the East Midlands Deanery. His career aspiration is to become a specialist in coronary and structural heart interventions. He is due to start a 2-year Interventional Cardiology Fellowship in London, Ontario, Canada, in July 2024.

## Supplementary Material

ytae333_Supplementary_Data

## Data Availability

No new data were generated or analysed in support of this research.

## References

[1] de Waard D, Alukayli M, Gelinas J, Iglesias I, Fujii S, Bagur R, et al Minimally invasive hybrid approach to high-risk mitral disease with severe mitral annular calcification. Can J Cardiol 2020;36:966.e11–966.e13. 10.1016/j.cjca.2019.12.008 32414620

[2] Ascione G, Denti P. Mitral annular calcification in patients with significant mitral valve disease: an old problem with new solutions. Front Cardiovasc Med 2022;9:1033565. 36479573 10.3389/fcvm.2022.1033565 PMC9719907

[3] Eleid MF, Wang DD, Pursnani A, Kodali SK, George I, Palacios I, et al 2-Year outcomes of transcatheter mitral valve replacement in patients with annular calcification, rings, and bioprostheses. J Am Coll Cardiol 2022;80:2171–2183. 36456047 10.1016/j.jacc.2022.09.037

[4] Vahanian A, Beyersdorf F, Praz F, Milojevic M, Baldus S, Bauersachs J, et al 2021 ESC/EACTS guidelines for the management of valvular heart disease. Eur Heart J 2022;43:561–632. 34453165 10.1093/eurheartj/ehab395

[5] Guerrero M, Vemulapalli S, Xiang Q, Wang DD, Eleid M, Cabalka AK, et al Thirty-day outcomes of transcatheter mitral valve replacement for degenerated mitral bioprostheses (valve-in-valve), failed surgical rings (valve-in-ring), and native valve with severe mitral annular calcification (valve-in-MAC) in the United States: data from the Society of Thoracic Surgeons/American College of Cardiology/Transcatheter Valve Therapy Registry. Circ Cardiovasc Interv 2020;13:e008425. 32138529 10.1161/CIRCINTERVENTIONS.119.008425

[6] Hatab T, Bou Chaaya RG, Zaid S, Wessly P, Satish P, Villanueva V, et al Feasibility and outcomes of mitral transcatheter edge-to-edge repair in patients with variable degrees of mitral annular calcification. J Am Heart Assoc 2023;12:e031118. 37753800 10.1161/JAHA.123.031118 PMC10727232

[7] Oezpeker UC, Barbieri F, Hoefer D, Bonaros N, Grimm M, Mueller L. Partial upper sternotomy is a safe alternative in mitral annulus decalcification. Semin Thorac Cardiovasc Surg 2022;34:502–509. 34089825 10.1053/j.semtcvs.2021.04.053

[8] Agrawal A, Reardon MJ, Goel SS. Transcatheter mitral valve replacement in patients with mitral annular calcification: a review. Heart Int 2023;17:19–26. 10.17925/HI.2023.17.1.19 PMC10339466 37456353

